# The prognostic significance of genetic polymorphisms *(Methylenetetrahydrofolate Reductase* C677T*, Methionine Synthase* A2756G*, Thymidilate Synthase* tandem repeat polymorphism) in multimodally treated oesophageal squamous cell carcinoma

**DOI:** 10.1038/sj.bjc.6602900

**Published:** 2005-12-06

**Authors:** M Sarbia, M Stahl, C von Weyhern, G Weirich, F Pühringer-Oppermann

**Affiliations:** 1 Department of Pathology, Technical University of Munich, Institut für Allgemeine Pathologie, Trogerstr. 28, Munich D-81675, Germany; 2 Department of Medical Oncology and Hematology, Kliniken Essen-Mitte, Essen University Medical School, Essen D-45136, Germany

**Keywords:** squamous cell carcinoma, oesophagus, genetic polymorphism, chemosensitivity

## Abstract

The present study retrospectively examined the correlation between the outcome of patients with locally advanced oesophageal squamous cell carcinoma (cT3-4 cN0-1 cM0) after multimodal treatment (radiochemotherapy±surgical resection), and the presence of genetic polymorphisms in genes involved in folate metabolism. In total, 68 patients who took part in a prospective multicentric trial received 5-fluorouracil (FU)-based radiochemotherapy, optionally followed by surgery. DNA was extracted from pretherapeutic tumour biopsies and was subsequently genotyped for common genetic polymorphisms of three genes (*MTHFR* C677T, *MTR* A2756G, *TS* tandem repeat polymorphism) involved in folate metabolism and potentially in sensitivity to 5-FU-based chemotherapy. The genotypes were correlated with tumour response to polychemotherapy, radiochemotherapy and with overall survival. Tumours with the *MTR* wild-type genotype (2756AA) showed a median survival time of 16 months, whereas tumours with an *MTR* variant genotype (2756AG/2756GG) showed a median survival time of 42 months (*P*=0.0463). No prognostic impact could be verified for the genotypes of the *MTHFR* genes and the *TS* gene. Among tumours treated with radiochemotherapy and subsequent resection, *MTR* variant genotype showed higher histopathological response rate than tumours with *MTR* wild-type genotype (*P*=0.0442). In contrast, no significant relationship between clinically determined tumour regression after polychemotherapy and polymorphisms of the three genes under analysis was observed. In conclusion, pretherapeutic determination of the *MTR* A2756G polymorphism may predict survival of multimodally treated oesophageal squamous cell carcinomas. Determination of *MTHFR* C677T and *TS* tandem repeat polymorphism has no predictive value.

In recent years, the prognosis of patients with oesophageal squamous cell carcinoma has only slightly improved. The results of surgical therapy have been poor, with 5-year survival rates varying between 9 and 40%, even in resectable stages (Enzinger and Mayer, 2003). Therefore, combined treatment modalities including chemotherapy, irradiation and surgical treatment were investigated in an increasing number of studies in order to improve survival of oesophageal cancer patients. These studies have indicated a complete response (CR) in 20–40% of patients preoperatively treated with combined radiotherapy and chemotherapy (Forastiere *et al*, 1997). However, concerning survival, proven benefit from combined neoadjuvant treatment modalities has been unequivocally shown only for the subset of patients showing a CR at histopathologic examination (Lerut *et al*, 1999; Ancona *et al*, 2001; Brücher *et al*, 2004). It may, therefore, be of great interest to find parameters that may help to identify those patients who will benefit from multimodal treatment modalities prior to the start of therapy (Forastiere *et al*, 1997; Lerut *et al*, 1999).

Methylenetetrahydrofolate reductase (MTHFR) and methionine synthase (MTR) are key enzymes in folate metabolism. The substrate for MTHFR, 5,10-methylene-tetrahydrofolate (THF) is used for conversion of dUMP to dTMP by the enzyme thymidylate synthase (TS). The enzyme product of MTHFR, 5-methyl-THF, serves as carbon donor for the remethylation of homocysteine to methionine, which is catalysed by the enzyme MTR (Figure 1) (Choi *et al*, 1998; Balley and Gregory, 1999). The *MTHFR* gene is highly polymorphic in the general population. The major nucleotide 677 polymorphism (C to T) results in an alanine to valine substitution, which alters enzyme activity (Goyette *et al*, 1994). Thus, individuals with the variant 677TT genotype have about 30% of the *in vitro* MTHFR enzyme activity of those with the 677CC wild-type genotype, whereas heterozygotes (677CT) have about 65% enzyme activity (Balley and Gregory, 1999). The *Methionine Synthase* (*MTR*) gene contains a common polymorphism at bp 2756 (*MTR* A2756G), which causes an amino-acid substitution from aspartic acid to glycine at codon 919 (D919G). Individuals with the 2756GG genotype have lower plasma homocysteine (or higher folate) levels than those with the 2756AA genotype (Tsai *et al*, 2000; Chen *et al*, 2001), indicating an increased enzymatic activity of the variant genotype.

An accumulation of 5,10-methylene-THF resulting from the *MTHFR* C677T polymorphism and/or the *MTR* A2756G polymorphism may have an effect on the response of cancer cells to 5-fluorouracil (5-FU), a chemotherapeutic agent frequently used in the neoadjuvant treatment of oesophageal squamous cell carcinoma. This is because 5-FU can form a ternary complex involving 5-fluoro-2′-deoyuridine-5′-monophosphate (5-FdUMP), TS, and 5,10-methylene-THF. The formation of this complex inhibits TS activity, which subsequently depletes intracellular thymidylate levels and ultimately suppresses DNA synthesis. Therefore, the *MTHFR* C677T polymorphism or the *MTR* A2756G polymorphism, which increase the intracellular concentrations of 5,10-methylene-THF, may increase the cytotoxic effect of 5-FU by increasing the formation of the 5,10-methylene-THF-TS-5-FdUMP ternary complex. The potential relevance of this mechanism has been recently shown *in vitro* where in colon cancer cell lines the *MTHFR* 677T polymorphism was associated with increased to sensitivity to 5-FU treatment (Sohn *et al*, 2004). Moreover, patients with advanced colorectal bearing the polymorphic *MTHFR* allele (either heterozygous or homozygous) responded better to 5-FU-based chemotherapy than patients with the wild-type *MTHFR* allele (Cohen *et al*, 2003). In contrast, the role of the *MTHFR* and *MTR* polymorphisms in 5-FU-treated oesophageal cancer has not been determined so far.

Additionally, the *TS* gene also contains a genetic polymorphism that may be also involved in efficacy of 5-FU-based chemotherapy. It is a 28-bp tandem repeat sequence within the 5′-untranslated region, and the vast majority of individuals show one of three genotypes, that is, two tandem repeats (2R/2R), three tandem repeats (3R/3R) or a heterozygous (2R/3R) genotype (Horie *et al*, 1995). This tandem repeat seems to function as an enhancer element since *in vitro* studies have shown that stepwise increase of *TS* mRNA expression and TS enzyme activity are associated with increasing number of tandem repeat sequences. Indeed, there is growing evidence that in colorectal cancer the TS 2R/2R genotype is associated with improved response and survival following 5-FU-based chemotherapy (Iacopetta *et al*, 2001; Villafranca *et al*, 2001; DiPaolo and Chu, 2004).

Therefore, in the present study, genotypes of *MTHFR*, *MTR* and *TS* were determined in preoperative biopsies derived from of multimodally treated patients with locally advanced oesophageal squamous cell carcinoma. Subsequently, these findings were correlated with response to 5-FU-based chemotherapy and overall survival.

## MATERIAL AND METHODS

### Patients

All patients of the present investigation were part of a prospective multicentric phase III study and gave written, informed consent to participate in this study. The results of this study, together with the criteria for patient selection and study design, have been extensively described elsewhere (Stahl *et al*, 1996, 2005).

### Patient selection and study design

In total, 68 patients with locally advanced oesophageal cancer (i.e. category T3/T4 according to the UICC classification (Sobin and Wittekind, 1997) defined by CT scan and endoscopic ultrasound), with or without regional lymph node metastases, were eligible.

Three courses of chemotherapy were administered within 9 weeks, followed by 4 weeks of radiotherapy with concomitant chemotherapy during the first 7 days. After the administration of chemotherapy and a cumulative dose of 40 Gy of radiotherapy, patients were either treated by at least further 25 Gy of radiotherapy (definitive radiochemotherapy) or by transthoracal oesophageal resection. Postoperative treatment was not performed.

### Preoperative chemotherapy and preoperative radiochemotherapy

Chemotherapy consisted of folinic acid 300 mg m^−2^, etoposide 100 mg m^−2^, bolus 5-FU 500 mg m^−2^, and cisplatin 30 mg m^−2^ on days 1–3, every 3 weeks (FLEP protocol).

Combined radiochemotherapy was administered on days 22–28 of the last course of chemotherapy. The oesophagus was irradiated using parallel-opposed anterior and posterior fields and photons from a 10- to 15-MV linear accelerator. A total dose of 40 Gy was given in daily fractions of 2 Gy, five times per week. During the first days of irradiation, the following chemotherapy was administered: cisplatin 50 mg m^−2^ on days 2 and 8, and etoposide 100 mg m^−2^ on days 4–6.

### Surgery

Resection of the oesophagus and the proximal stomach was performed by a combined right thoracal and abdominal approach. Resection included excision of the paraoesophageal, paracardial, left gastric, and celiac lymph nodes.

### Criteria for response to chemotherapy and radiochemotherapy

Response to chemotherapy was evaluated clinically after the third course and included barium oesophagogram, oesophagoscopy, and computed tomography of the chest and abdomen. Response was categorised as follows: CR – normal barium oesophagogram, no visible tumour by oesophagoscopy, biopsies free of tumour tissue, and normal CT; partial response (PR) – greater than 50% tumour regression as evaluated by CT, and greater than 50% reduction of intraoesophageal tumour extension as assessed by barium swallow and oesophagoscopy; no change (NC) – less than 50% regression of tumour extension, and no evidence of tumour progression; progressive disease (PD) – increasing tumour obstruction indicated by barium swallow or oesophagoscopy, and increasing tumour diameter assessed by CT.

Response to radiochemotherapy was evaluated by histopathological examination of cases that underwent transthoracal oesophageal resection. In this evaluation, complete responders (no viable tumour cells in the resection specimens) were separated from cases were incomplete or no tumour regression was detectable.

### Pathologic review of pretherapeutic tumour biopsies

Tumour biopsies that had been endoscopically obtained during pretherapeutic staging procedures were retrieved from the files of pathologic institutes associated with the medical centers that took part in this study. Of the 68 patients, 60 were male and eight were female. The median age was 57 years (range: 37–70). A total of 58 tumours had been clinically categorised as T3 and 10 tumours had been categorised as T4; 15 cases were in category N0 and 53 cases were in category N1. Histologic slides of the biopsy specimens were stained with hematoxylin and eosin for the determination of tumour type and tumour grade according to the WHO criteria. Three tumours were graded as G1, 34 tumours were graded as G2, and 31 tumours were graded as G3.

### Genotyping of MTHFR C677T, MTR A2756G and TS

For the determination of individual genotypes, genomic DNA was extracted from formaldehyde-fixed tumour biopsies using commercially available DNA-preparation kits (Qiagen, Hilden, Germany). The *MTHFR* C677T genotype was determined by real-time fluorescence PCR and melting curve analysis using a LightCycler instrument (Roche Diagnostics, Mannheim, Germany). PCR conditions, primers and probes and melting curve analysis were applied as previously described (Sarbia *et al*, 2005). A melting curve with a single peak at low melting temperature (50°C) indicates a homozygous variant (677TT) genotype, a single peak at high melting temperature (60°C) indicates a homozygous wild-type (677CC) genotype, and a double peak indicates a heterozygous genotype.

A PCR-RFLP assay was used to determine the *MTR* A2756G genotype. The primers were forward 5′-GAA CTA GAA GAC AGA AAT TCT CTA-3′ and reverse 5′-CAT GGA AGA ATA TGA AGA TAT TAG A-3′ (Leclerc *et al*, 1996). The PCR product is digested with *Hae*III (New England Biolabs, Beverly, MA, USA); and the amplified fragment of 189 bp is cut into fragments of 159 and 30 bp in the presence of the G allele.

The promoter region of the TS gene was amplified by PCR using the following primers: forward 5′-AAAAGGCGCGCGGAAGGGGTCCT-3′ and reverse 5′-TCCGAGCCGGCCACCAGGCAT-3′ (Iacopetta *et al*, 2001). The PCR products were analysed by length differences using ethidium-bromide-stained 2% agarose gels.

### Statistical analysis

SAS software package (SAS Institute Inc., Carey, NC, USA) was used for statistical analyses. Statistical analysis of the correlation between tumour regression following chemotherapy and genotypes (*MTHFR*, *MTR*, *TS*) was performed by means of the two-tailed Fisher's exact test. Therefore, the parameters were dichotomised as follows: tumour response (CR/PR *vs* NC/PD), *MTHFR* (CC *vs* CT/TT), *MTR* (AA *vs* AG/GG) and *TS* (2R/2R *vs* 2R/3R/3R/3R). The Kaplan–Meier method for analysis of censored data was used for calculating survival rates and tested with the log-rank test and the Wilcoxon test. Probabilities <0.05 were regarded as statistically significant.

## RESULTS

### Correlation between genotypes and tumour regression following chemotherapy and radiochemotherapy

Following FLEP chemotherapy, three patients (4.4%) received CR, 28 PR (41.2%), 32 NC (47.1%) and five PD (7.3%). Data on histopathological response evaluation after radiochemotherapy was available only in a subset of patients that underwent transthoracal oesophageal resection (*n*=20). Among these cases, seven (35.0%) showed complete regression without viable residual tumour cells, whereas in the other 13 (65.0%) cases either incomplete regression or no histological signs of tumour regression were present.

In total, 67 samples could be genotyped successfully for the *MTHFR* gene (98.5%), 64 for the *MTR* gene (94.1%) and 57 for the *TS* gene (83.8%).

No significant relationship between tumour regression after FLEP chemotherapy and polymorphisms of the three genes under analysis was observed (Table 1). In contrast, response to radiochemotherapy was correlated with the *MTR* gene polymorphism. Tumours with an *MTR* variant genotype (2756AG/2756GG) showed complete histopathological regression in 46.2% of cases whereas tumours with the *MTR* wild-type genotype (2756AA) showed complete regression in 0% of cases (Table 2). (*P*=0.0442 according to the *χ*^2^ test). No correlation between response to radiochemotherapy and *MTHFR* and *TS* genotypes was found.

### Survival analysis

The overall survival of all 68 patients has been followed up every 3 months for the first 3 years after the end of treatment; afterwards every 6 months. One patient was lost to follow-up. At the end of the follow-up period (30.4.2005), 14 of the remaining 67 patients (20.9%) were still alive. The follow-up time for all 67 patients ranged from 4 to 114 months (median: 22 months). The follow-up time for the 14 patients at risk ranged from 29 to 114 months (median: 83 months).

**Figure 2:**
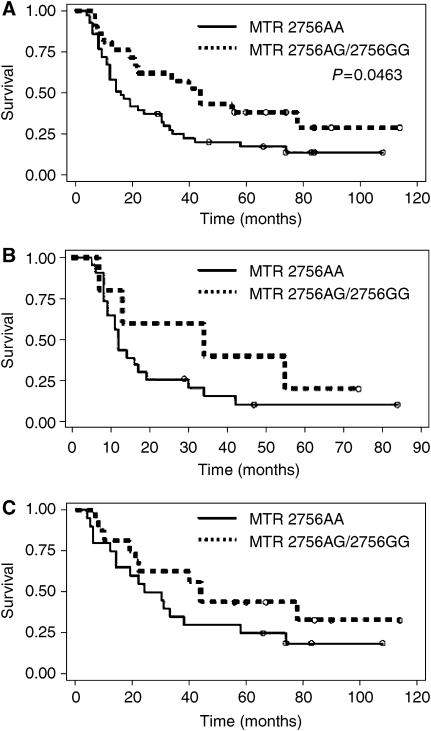
Survival curves of oesophageal SCC patients treated either by neodjuvant radiochemotherapy and subsequent resection or by definitive radiochemotherapy in relation Methionine Synthase genotype (*n*=63, **A**). Subgroup analysis for patients either treated by neoadjuvant radiochemotherapy and resection (*n*=28, **B**) or by definitive radiochemotherapy (*n*=35, **C**).

All patients received complete treatment. Of the 67 patients suitable for survival analyses, 38 received definitive radiochemotherapy and 29 were treated by radiochemotherapy and subsequent oesophageal resection. Analysing all 67 patients, tumours with the *MTR* wild-type genotype (2756AA) showed a median survival time of 16 months whereas tumours with an *MTR* variant genotype (2756AG/2756GG) showed a median survival time of 42 months (Figure 2) (*P*=0.0463 according to the Wilcoxon test; *P*=0.566 according to the log-rank test).

No prognostic impact could be verified for the genotypes of the *MTHFR* genes and the *TS* gene either in the group as a whole (Table 3) or when stratified, according to the type of therapy (radiochemotherapy followed by resection or definitive radiochemotherapy).

## DISCUSSION

The current study indicates that multimodally treated oesophageal squamous cell carcinoma patients with the *MTR* 2756AG/2756GG genotypes may have better survival than individuals with the *MTR* 2756AA genotype. Mechanistically, this may make sense taking into account the putative effect of this polymorphism on folate metabolism. Thus, *MTR* catalyses the transfer of the methyl base from 5-methyl THF to homocysteine, thereby producing methionine and THF. THF is the precursor of 5′-formyl THF, which is an inhibitor of thymidylate synthase in cooperation with 5′-FdUMP, which is a metabolite of 5′-FU. Therefore, an increased activity of the *MTR* gene may lead to an accumulation of 5′-formyl THF and thereby to a pronounced cytotoxic effect of 5′-FU treatment. The *MTR* 2756GG polymorphism was initially thought to be associated with lower enzyme activity than the *MTR* 2756AA genotype (Leclerc *et al*, 1996). However, more recent investigations indicate that the variant genotypes (2756AG, 2756GG) may be associated with lower homocysteine and/or higher methionine levels than the wild-type genotype, implying a more effective enzyme activity (Tsai *et al*, 2000; Chen *et al*, 2001; Silaste *et al*, 2001).

On the other hand, we did not observe any effect of the *MTHFR* C677T polymorphism on survival. If the hypothesis is true that accumulation of 5′-formyl THF increases the effect of 5′-FU-based therapy, this observation is difficult to understand. This is because the variant genotype of the *MTHFR* gene is characterised by less effective catalyzation of 5′10′-methylene THF to 5′methyl THF and thus to an accumulation of 5′-formyl THF. However, one clearly has to take into account that a large variety of other factors than genetic polymorphisms may influence the activity of the key enzymes involved in folate metabolism. Among others, enzyme activities may be influenced by transcriptional regulation, post-transcriptional modification and protein modification. Moreover, 5-FU was not the only cytotoxic drug used in the chemotherapy protocol of the current study. Therefore, the putative effect of 5-FU-associated polymorphisms may be superposed by other effects. Therefore, currently, only empirical studies like the present one may clarify whether a given molecular aberration is associated with clinically important effects or not. Nonetheless, determination of genetic polymorphisms, for example, in peripheral blood, is considered an attractive approach for response prediction because this represents a relatively noninvasive approach for molecular profiling. Other methods, for example, immunohistochemistry and RT-PCR based on endoscopically obtained biopsies, always have the inherent doubt of representativity for the entire tumour. On the other hand, a recent study indicated that determination of genetic polymorphisms in peripheral blood may not be representative for the situation in tumour tissue. Thus, Uchida *et al* (2004) have shown that individuals heterozygous for the 28-bp polymorphism in the *TS* gene may have cancers that are homozygous for this polymorphism due to loss of one allele during carcinogenesis. Furthermore, they could show that the response to 5-FU-based chemotherapy in these cases was comparable to cases where the entire individual was homozygous. Therefore, it may be superior to analyse the genotype of polymorphisms in tumour cells than in peripheral blood.

In conclusion, our data indicate that determination of the *Methylenetetrahydrofolate Reductase C677T* polymorphism and the *Thymidilate Synthase* tandem repeat polymorphism does not provide significant predictive information in multimodally treated oesophageal squamous cell cancer patients. The potential effect of the *Methionine Synthase* A2756G polymorphism deserves further investigations in similar designed studies.

## Figures and Tables

**Figure 1:**
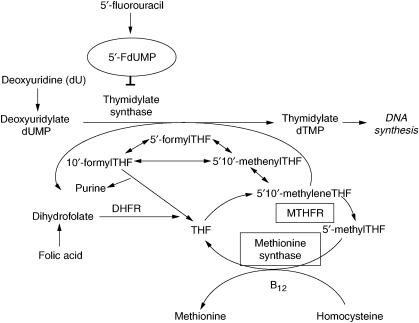
Schematic representation of the catalytic activity of the three enzymes under investigation (Methionine Synthase, Thymidylate Synthase, Methylenetetrahydrofolate Reductase (MTHFR)).

**Table 1:** Correlation between *MTHFR*, *MTR* and *TS* genotypes and clinically determined tumour regression following polychemotherapy in multimodally treated oesophageal SCC^a^

**Genotypes**	**Complete remission/partial remission (%)**	**No change/progression (%)**
*MTHFR* 677CC	12 (46.2)	14 (53.8)
*MTHFR* 677CT/677TT	19 (46.3)	22 (53.7)
		
*MTR* 2756AA	20 (45.5)	24 (54.5)
*MTR* 2756AG/2756GG	9 (45.0)	11 (55.0)
		
*TS* 2R/2R	15 (45.5)	18 (54.5)
*TS* 2R/3R/3R/3R	12 (50.0)	12 (50.0)

a No significant correlations according to *χ*^2^ test.

**Table 2:** Correlation between MTHFR, MTR and TS genotypes and tumour regression as determined by histopathological examination following radiochemotherapy in multimodally treated oesophageal SCC

**Genotypes**	**Complete histopathological response (%)**	**Incomplete histopathological response/no change/progression (%)**	***P*-value**
*MTHFR* 677CC	3 (33.3)	6 (66.7)	NS^a^
*MTHFR* 677CT/677TT	4 (40.0)	6 (60.0)	
			
*MTR* 2756AA	0 (0)	6 (100)	*P*=0.0442
*MTR* 2756AG/2756GG	6 (46.2)	7 (53.8)	
			
*TS* 2R/2R	4 (40.0)	6 (60.0)	NS
*TS* 2R/3R/3R/3R	2 (28.6)	5 (71.4)	

a No significant correlations according to *χ*^2^ test.

**Table 3:** Correlation between *MTHFR*, *MTR* and *TS* genotypes and overall survival in multimodally treated oesophageal SCC

**Therapy**	**Genotypes**	***n***	**Censored (%)**	**Median survival (months)**	***P*-value^a^**
All patients	*MTHFR* 677CC	25	4 (16%)	19	NS^b^
	*MTHFR* 677CT/677TT	41	10 (24%)	30	
RCTx^c^+resection	*MTHFR* 677CC	13	1 (8%)	12	NS
	*MTHFR* 677CT/677TT	15	4 (27%)	16	
Definitive RCTx	*MTHFR* 677CC	12	3 (25%)	55	NS
	*MTHFR* 677CT/677TT	26	6 (23%)	30	
					
All patients	*MTR* 2756AA	43	7 (16%)	16	*P*=0.0463
	*MTR* 2756AG/2756GG	20	6 (30%)	42	
RCTx+resection	*MTR* 2756AA	23	3 (13%)	12	NS
	*MTR* 2756AG/2756GG	5	1 (20%)	34	
Definitive RCTx	*MTR* 2756AA	20	4 (20%)	14	NS
	*MTR* 2756AG/2756GG	15	5 (33%)	21	
					
All patients	*TS* 2R/2R	32	7 (22%)	17	NS
	*TS* 2R/3R/3R/3R	24	5 (21%)	26	
RCTx+resection	*TS* 2R/2R	13	1 (8%)	9	NS
	*TS* 2R/3R/3R/3R	10	3 (30%)	24	
Definitive RCTx	*TS* 2R/2R	19	6 (32%)	31	NS
	*TS* 2R/3R/3R/3R	14	2 (14%)	26	

a According to the Wilcoxon test.

b Not significant.

c Radiochemotherapy.
